# Spatio-temporal modelling of dengue counts in the Central Valley of Costa Rica

**DOI:** 10.1017/S0950268826101204

**Published:** 2026-02-20

**Authors:** Cathy W. S. Chen, Shu Wei Chou-Chen, Hsiao-Hsuan Liao

**Affiliations:** 1 Department of Statistics, Feng Chia University, Taiwan; 2 School of Statistics & Center for Pure and Applied Mathematics Research, University of Costa Rica, Costa Rica

**Keywords:** dengue case counts, endemic–epidemic model, Fourier seasonality, integer-valued GARCH models, spatio-temporal modelling

## Abstract

This study analyses 18 years of weekly reported dengue cases (January 2002–December 2020; 988 weeks) from Costa Rica’s Central Valley to examine seasonal and multi-year patterns. To model the spatio-temporal dynamics of dengue, we employ three statistical approaches for case counts: the spatial hurdle integer-valued generalized autoregressive conditional heteroskedasticity (INGARCH) model, the spatial zero-inflated generalized Poisson (ZIGP)-INGARCH model, and the endemic–epidemic (EE) model. Covariates include rainfall and maximum temperature or alternatively seasonal Fourier terms to represent annual seasonality. Using a Bayesian framework, we fit the spatial INGARCH-family models to weekly dengue cases. The EE model and the ZIGP-INGARCH model, both with Fourier seasonal terms, show the best predictive accuracy and provide estimates of seasonal intensity and peak timing relevant for dengue surveillance. Incorporating annual seasonality improves modelling of multivariate weekly dengue cases in Costa Rica’s Central Valley, underscoring the importance of cyclical patterns for strengthening early warning systems and guiding targeted vector control.

## Introduction

Dengue is an infectious disease with both endemic and epidemic characteristics, transmitted primarily by the mosquito species *Aedes aegypti* and *Aedes albopictus.* It is the most prevalent arboviral disease worldwide, imposing significant burdens in terms of morbidity and economic impact [1]. Costa Rica, a Central American country with an area of 51,179 km^2^ and diverse microclimates, has faced a persistent challenge from dengue in its public health system. After authorities certified the eradication of the vector *A. aegypti* in 1955, inconsistent health surveillance allowed the virus to reenter Costa Rica, leading to the reappearance of cases in 1993. Since then, the Costa Rican Ministry of Health reported a total of 398,546 dengue cases in Costa Rica between 1993 and 2021 [2]. Despite preventive measures, controlling the spread of the disease remains challenging. Consequently, there is increasing interest in adopting more strategic approaches to prevention and response, aiming to reduce case numbers and enhance the management of medical resources.

In Costa Rica, dengue is transmitted mainly by *A. aegypti*, with *A. albopictus* playing a secondary role. Female mosquitoes usually stay near their emergence sites, flying up to 400 m to find egg-laying locations [3]. While mosquito dispersal is relatively limited, humans play a crucial role in the spread of the virus, carrying it to new areas during the infectious phase. This human-mediated movement facilitates the introduction of dengue into previously unaffected locations, underscoring the importance of examining its spatio-temporal transmission patterns.

To characterize the spatio-temporal dynamics of dengue transmission, accounting for excess zeros, overdispersion, geographic clustering, and delayed effects, we apply three complementary statistical models: (i) the spatial hurdle integer-valued generalized autoregressive conditional heteroskedasticity (INGARCH) model, (ii) the spatial zero-inflated generalized Poisson (ZIGP)-INGARCH model [3] and its recent extension [4], and (iii) the endemic–epidemic (EE) model [5, 6].

The spatial hurdle-INGARCH model accommodates overdispersion and excess zeros by modelling the occurrence and intensity processes separately, while the spatial ZIGP-INGARCH model addresses excess zeros and varying dispersion within a unified likelihood structure. Both models use information from neighbouring areas, reflecting the interconnected nature of space and time and allowing joint modelling of count dynamics across municipalities. The EE model also incorporates spatio-temporal dependence but decomposes incidence into an endemic baseline and an epidemic component, representing persistent presence and short-term transmission within a unified framework.

Dengue incidence is closely linked to weather patterns and climatological conditions [7–9]. In Costa Rica, several studies examine this relationship using municipality-level surveillance data. For example, [10, 11] develop predictive models based on generalized additive models, random forests, and generalized additive models for location, scale, and shape (GAMLSS) to estimate weekly dengue relative risks separately for each of the 32 municipalities. More recently, [12] employs a Bayesian spatio-temporal framework implemented via Integrated Nested Laplace Approximation (INLA) to analyse and predict monthly dengue counts and relative risks for the same set of municipalities. Although spatial dependence is incorporated, the use of monthly aggregation simplifies the temporal structure and limits the resolution of short-term transmission dynamics. In contrast, the present study focuses on weekly dengue counts and explicitly incorporates spatial effects within the Central Valley of Costa Rica, which allows a more detailed examination of seasonal dynamics and short-term fluctuations associated with climatic and environmental drivers.

Within this framework, rainfall and temperature act as key environmental drivers shaping vector ecology and the intensity of dengue transmission. Rainfall influences the availability and persistence of mosquito breeding sites and larval survival, while temperature governs mosquito development rates, survival, and viral replication within the vector. Although these climatic factors underpin recurrent seasonal patterns in dengue incidence, their effects may be nonlinear and operate with temporal delays, which can limit their short-horizon predictive contribution when included directly as covariates.

Motivated by these mechanisms, our study focuses on weekly dengue data and incorporates spatial effects in the Central Valley of Costa Rica, a region characterized by relatively homogeneous climatic conditions and strong spatial interaction between municipalities. Compared with monthly data, modelling dengue counts at a weekly scale introduces additional complexity, as weekly series exhibit stronger short-term fluctuations, higher variability, and more pronounced zero inflation, which can obscure underlying trends and amplify noise. Although weekly aggregation mitigates many reporting delays present in daily data, some irregularities in surveillance data may still remain. Building on these considerations, our study incorporates meteorological covariates and seasonal components to capture climate variability and recurring patterns in dengue transmission.

This study advances dengue surveillance research by systematically comparing spatio-temporal count models under alternative representations of seasonality, using a long weekly dataset from multiple municipalities within a climatically homogeneous region. Rather than relying on a single seasonal formulation, we examine models based on climatic covariates, Fourier seasonal terms, and their combinations, thereby evaluating how different approaches capture seasonal dynamics in dengue transmission. By focusing on geographically proximate municipalities with similar climatic regimes, we assess when parsimonious Fourier seasonal factors can effectively summarize both observed climatic influences and other unmeasured seasonal drivers. This comparative framework provides practical guidance for selecting seasonal components in applied infectious disease modelling and supports the development of robust early warning systems.

In addition, the study provides a clear conceptual clarification of how spatial interaction is represented across commonly used modelling frameworks, highlighting differences between adjacency-based weighting in the EE model and distance-based weighting in INGARCH-family models. This clarification supports appropriate model choice and interpretation in applied spatial epidemiological studies.

## Data description

Costa Rica exhibits a wide range of microclimates across its seven distinct climate regions, each defined by distinct climatic and seasonal patterns [13]. A region of particular interest in Costa Rica is the Central Valley, which has a tropical rainforest climate based on the Köppen–Geiger classification. Moreover, this area has the highest population density, urbanization level, socioeconomic activity, and healthcare standards. This study focuses on modelling four municipalities within the Central Valley: San José, Alajuelita, Desamparados, and Santa Ana, which are among the 32 municipalities identified by the Ministry of Health and previous studies as having the highest incidence of dengue cases.

**Figure 1.**
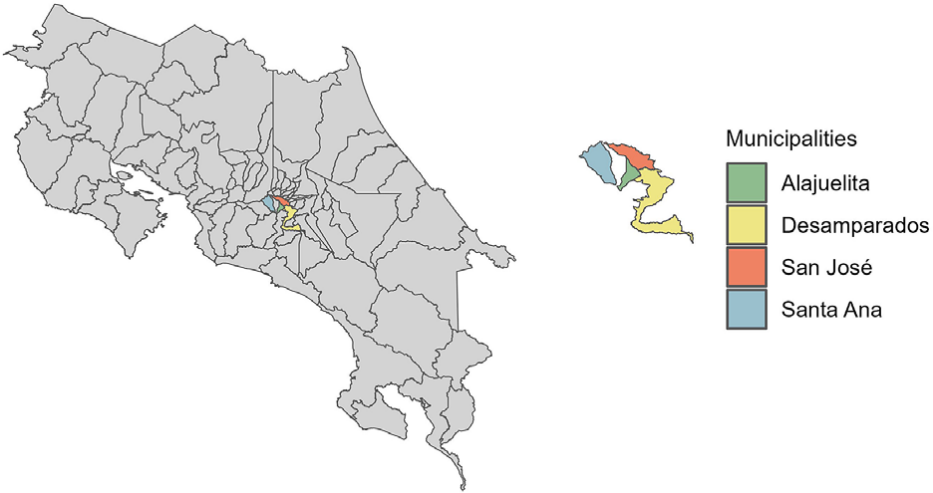
Locations of the four municipalities in Costa Rica.

**Figure 2.**
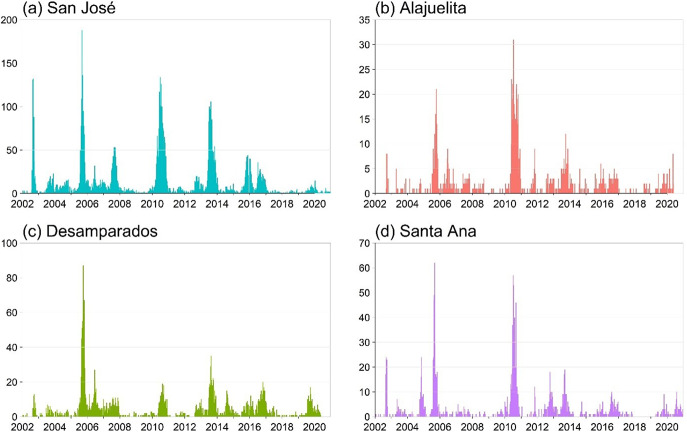
Weekly dengue case counts for the four municipalities, 2002–2021, with vertical stems indicating the number of cases.

The dataset from the Ministry of Health of Costa Rica spans January 2002 to December 2020, totalling 988 weeks. Figure 1 displays the geographic locations of the four municipalities in the Central Valley analyzed in this study, and Figure 2 presents the weekly dengue case counts as vertical stems. Table 1 provides the coordinates of the geographical centre for each municipality, which are used to construct the spatial components of the INGARCH-type models. The summary statistics in Table 2 reveal substantial heterogeneity in weekly dengue cases across municipalities in Costa Rica’s Central Valley. San José has the highest mean and variance, reflecting greater intensity and variability in outbreaks, whereas Alajuelita and Santa Ana exhibit low means but high proportions of zero observations.

**Table 1.** Geographical coordinates of the four municipalities in Costa Rica’s Central Valley

Costa Rica	Municipality	Longitude	Latitude
	San José	−84.07781	9.93551
	Alajuelita	−84.09927	9.90283
	Desamparados	−84.06490	9.89784
	Santa Ana	−84.18039	9.93244

**Table 2.** Summary statistics of weekly dengue cases for each municipality in Costa Rica’s Central Valley.

Municipality	Weekly dengue cases						
	Min	Median	Max	Mean	Variance	Dispersion ratio	Percentage of zeros
San José	0	3	188	10.633	443.355	41.698	19.74%
Alajuelita	0	0	31	1.325	9.778	7.38	60.43%
Desamparados	0	1	87	3.324	45.504	13.69	38.26%
Santa Ana	0	0	62	2.077	32.576	15.685	54.25%

**Figure 3.**
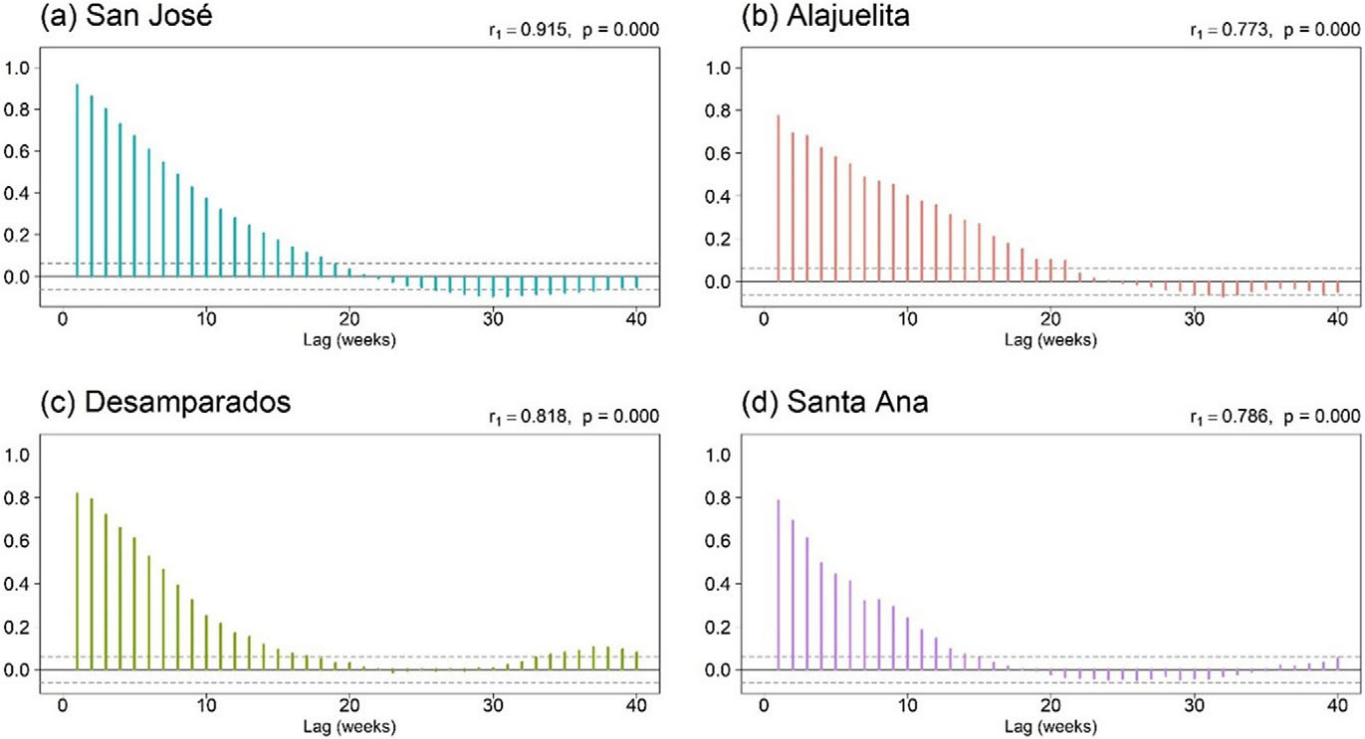
Autocorrelation function plots of weekly dengue case counts for the four municipalities in Costa Rica’s Central Valley.

**Figure 4.**
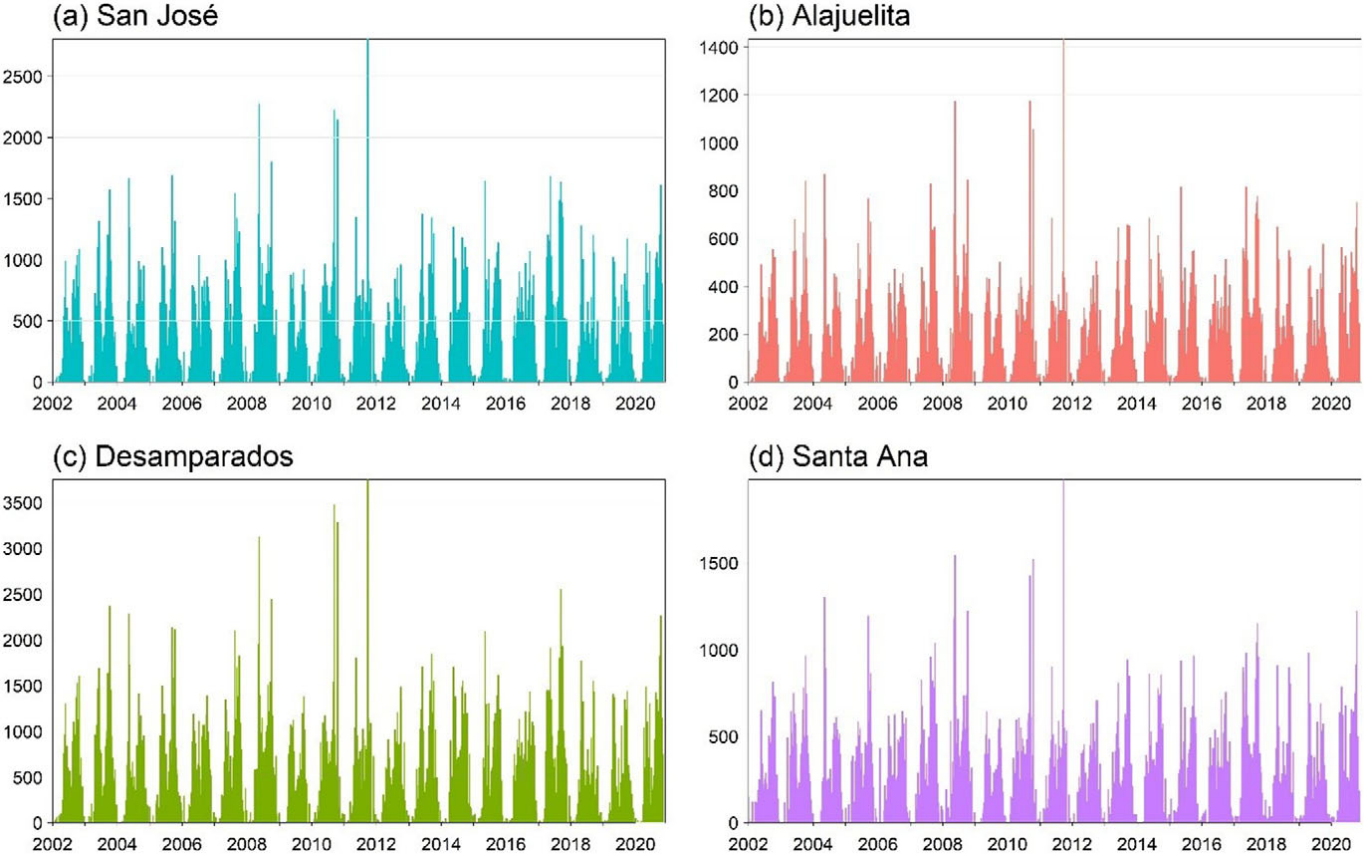
Weekly rainfall totals for San José, Alajuelita, Desamparados, and Santa Ana, 2002–2020.

**Figure 5.**
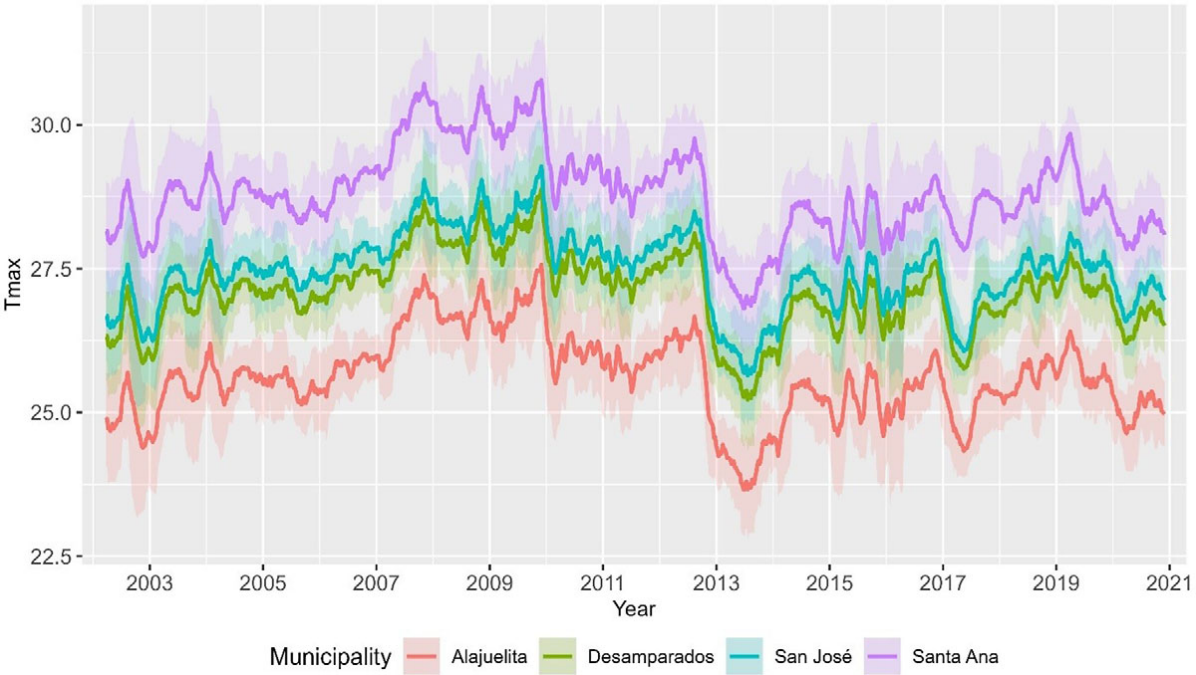
Weekly maximum temperature for San José, Alajuelita, Desamparados, and Santa Ana, 2002–2020, shown as a 13-week rolling mean with ±1 SD ribbons.

To assess temporal and spatial dependence, Figure 3 displays the short lag autocorrelation for each municipality, indicating week-to-week persistence in dengue activity. The decay rate varies by municipality, which supports models that include serial dependence in the counts. Figure 4 displays weekly rainfall as vertical stems, where peaks and dry spells are readily identifiable with similar timing across municipalities but differing magnitudes by site. Figure 5 shows weekly maximum temperature as a 13-week rolling mean with one-standard-deviation bands for the four municipalities. The series follow similar temporal patterns, while the width of the shaded bands reflects short-term variability. We apply a shifted standardization to each climate covariate, with 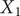 denoting rainfall and 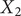 denoting maximum temperature. Specifically, for each series, we subtract its sample minimum and then divide by its standard deviation. This transformation reduces the influence of large magnitudes, preserves nonnegativity, and places the covariates on comparable scales. Precipitation data come from the Climate Hazards Group InfraRed Precipitation with Station data (CHIRPS) [14], and temperature data come from the Geophysical Research Center (CIGEFI) of the University of Costa Rica. Both datasets have a spatial resolution of 5 km 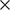 5 km and are aggregated to the municipality level. As covariates, we consider two climate variables, rainfall 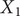 and maximum temperature 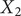 . Seasonal effects are represented either through these climate variables or through seasonal Fourier terms defined as 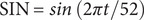 and 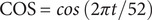 . In some model specifications, both climate variables and Fourier terms are included.

## Methodology

This study adopts the spatial ZIGP-INGARCH approach [4] to capture excess zeros, overdispersion, temporal dependence, and spatial correlation, while incorporating covariates to improve model calibration. Applied to weekly dengue counts, let 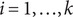 index the 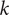 municipalities and 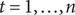 index weeks. The model assumes conditional independence across municipalities given past information 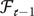 , with each 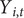 following a ZIGP distribution:(1)

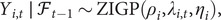


(2)

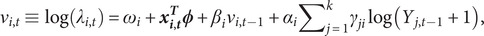

where 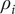 is the probability of excess zeros, 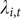 is the conditional mean (or intensity) parameter, 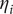 is the dispersion parameter, 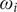 , 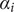 , 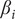 , and 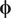 are unknown parameters, 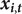 is a vector of exogenous covariates, and 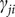 denotes the spatial weights capturing the influence of cases in location *j* on location *i.* To emphasize strong dependence and ensure stationarity, we impose the following constraints on the coefficients 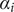 and 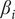 in Equation (2): 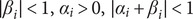 .

Regarding the spatial component 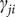 , we use the Euclidean distance 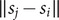 to measure the geographical separation between municipalities *i* and *j.* Let 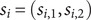 denote the geographical coordinates (longitude and latitude) of municipality *i*, where *i* = 1, *…, k.* To represent the influence of dengue cases from one municipality on another, we construct a spatial weight 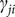 based on the geographical distance between municipalities *i* and *j.* First, we calculate the Euclidean distance between their geographical centres as

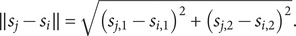



This distance is then converted into a measure of spatial closeness:

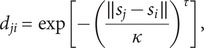

where 0 *≤ τ* < 2 controls the shape of the distance decay relationship and *κ* > 0 is a scaling parameter, both treated as unknown parameters to be estimated from the data. Finally, the spatial weight is normalized so that the weights from all other municipalities to *i* sum to 1:

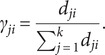

Higher values of *γ_ji_* indicate stronger spatial influence from municipality *j* on municipality *i.*

The spatial hurdle-INGARCH specification [4] assumes that the conditional distributions of *Y*_*i*,*t*_, for *i* = 1, *…*, *k*, given 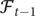 , are independent, with each following(3)

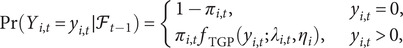


(4)

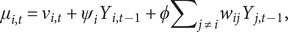

where 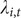 is defined in Equation (2), 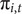 is the probability of a positive count at municipality *i* and time *t*, and 
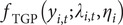
 denotes the probability mass function of the truncated generalized Poisson distribution, with conditional mean 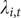 and dispersion parameter *η_i_.*

We also consider the extended framework of the EE model [15] for comparison. Although the EE framework decomposes incidence into endemic and epidemic components and models how cases in one region can influence cases in other regions over time, it can also be viewed as a multivariate count time series model. For *k* municipalities, the model assumes

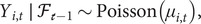


(5)

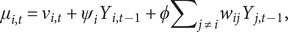




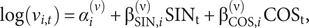

where 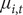 is the conditional mean; 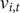 is the endemic component with municipality-specific intercept and covariates; 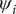 is the municipality-specific autoregressive epidemic parameter; and 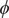 is the common neighbourhood epidemic parameter weighted by 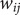 . The Poisson distribution can be replaced by a negative binomial NB 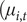 , 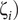 , where 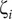 >0 is the location-specific overdispersion parameter. The weights are row-standardized so that the total of all 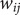 for 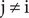 equals 1, making the neighbourhood effect the average influence from adjacent municipalities. In contrast to this adjacency-based specification in the EE model, the ZIGP-INGARCH and hurdle-INGARCH models incorporate spatial dependence through distance-based weights that allow spatial influence to vary smoothly with geographical distance.

Many studies have successfully modelled spatio-temporal processes using the Bayesian approach (see, for example, [12]). In the present study, we apply Bayesian Markov Chain Monte Carlo (MCMC) methods to fit the spatial ZIGP-INGARCH and hurdle-INGARCH models, and we estimate the EE model under a maximum likelihood framework using the hhh4addon package in R.

## Results and discussion

To capture recurrent seasonal patterns that summarize the combined influence of climatic and other unobserved drivers, we model seasonality using either climatic covariates (X_1_: rainfall; X_2_: maximum temperature) or sine and cosine Fourier terms. Accordingly, seasonality is specified using one of four approaches: a purely spatio-temporal model without covariates, a model with climatic covariates only, a model with Fourier terms only, or a model with climatic covariates augmented by a Fourier component. Table 3 compares the predictive accuracy of 16 models across four municipalities using mean-square error (MSE) and pooled root MSE (RMSE), both of which are widely used measures of forecast accuracy. The pooled RMSE is computed from pooled squared prediction errors across municipalities. The EE model with (SIN, COS) achieves the lowest pooled RMSE (1.779) when using truncated mean predictions (*μ*_*i*,*t*_) for comparison, whereas all spatial models directly produce count predictions. To ensure fairness, we treat the competing models as two groups, those based on conditional means and those based on prediction counts, and select the best-performing model from each.

**Table 3.** Model comparison based on MSE and pooled RMSE for the four municipalities

Model	San José	Alajuelita	Desamparados	Santa Ana	Total MSE	Pooled RMSE
hurdle	34.6354	2.1628	6.4619	6.1364	49.3964	3.5141
hurdle + SIN	28.7079	1.7759	5.0254	5.1957	40.7049	3.1900
hurdle + COS	26.6805	2.0122	6.3783	5.5953	40.6663	3.1885
hurdle + SIN COS	23.9168	1.7637	5.6501	4.5598	35.8905	2.9954
hurdle + (*X*_1_, *X*_2_)	32.2617	1.8651	5.0669	6.0325	45.2262	3.3625
hurdle + (*X*_1_, *X*_2_) + SIN	29.5558	1.7373	4.6765	5.0984	41.0680	3.2042
hurdle + (*X*_1_, *X*_2_) + COS	27.0791	1.7972	5.5903	5.3935	39.8600	3.1567
ZIGP	18.9250	3.2576	6.9331	6.2454	35.3611	2.9733
ZIGP + SIN	17.9990	2.2850	4.9391	4.4635	29.6866	2.7243
ZIGP + COS	17.8813	2.6156	6.7465	5.1460	32.3895	2.8456
ZIGP + SIN COS	16.1866	2.2972	5.5112	4.1207	**28.1156**	**2.6512**
ZIGP + (*X*_1_, *X*_2_)	19.0497	2.3185	5.4533	5.4594	32.2809	2.8408
ZIGP + (*X*_1_, *X*_2_) + SIN	18.1248	2.2373	4.8763	4.5477	29.7860	2.7288
ZIGP + (*X*_1_, *X*_2_) + COS	17.7323	2.2941	6.0101	5.1075	31.1440	2.7903
EE_PO_ + SIN COS	4.8967	2.1064	3.3222	2.3293	**12.6545**	**1.7787**
EE_NB_ + SIN COS	17.4063	2.7781	4.5431	4.5552	29.2827	2.7057

*Note: X*_1_: rainfall; *X*_2_: maximum temperature. EE_PO_: Poisson; EE_NB_: negative binomial; both evaluated using conditional means. Bold values indicate the smallest (best) performance metrics within each model class, evaluated separately for spatial INGARCH-type models and EE models.

**Figure 6.**
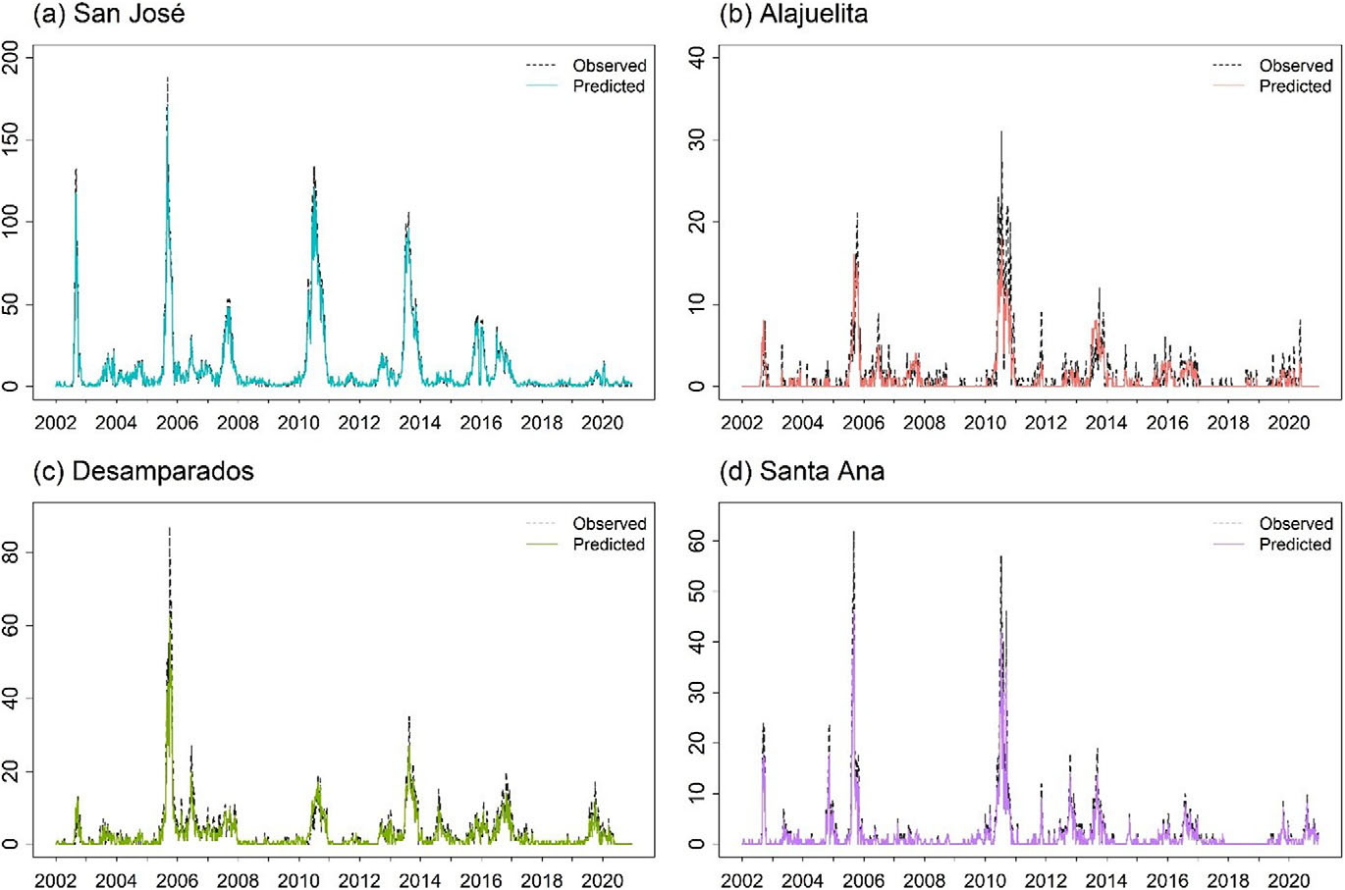
Mean predictions of weekly dengue case counts for the four municipalities, 2002–2020, based on the EE model with (SIN, COS).

**Figure 7.**
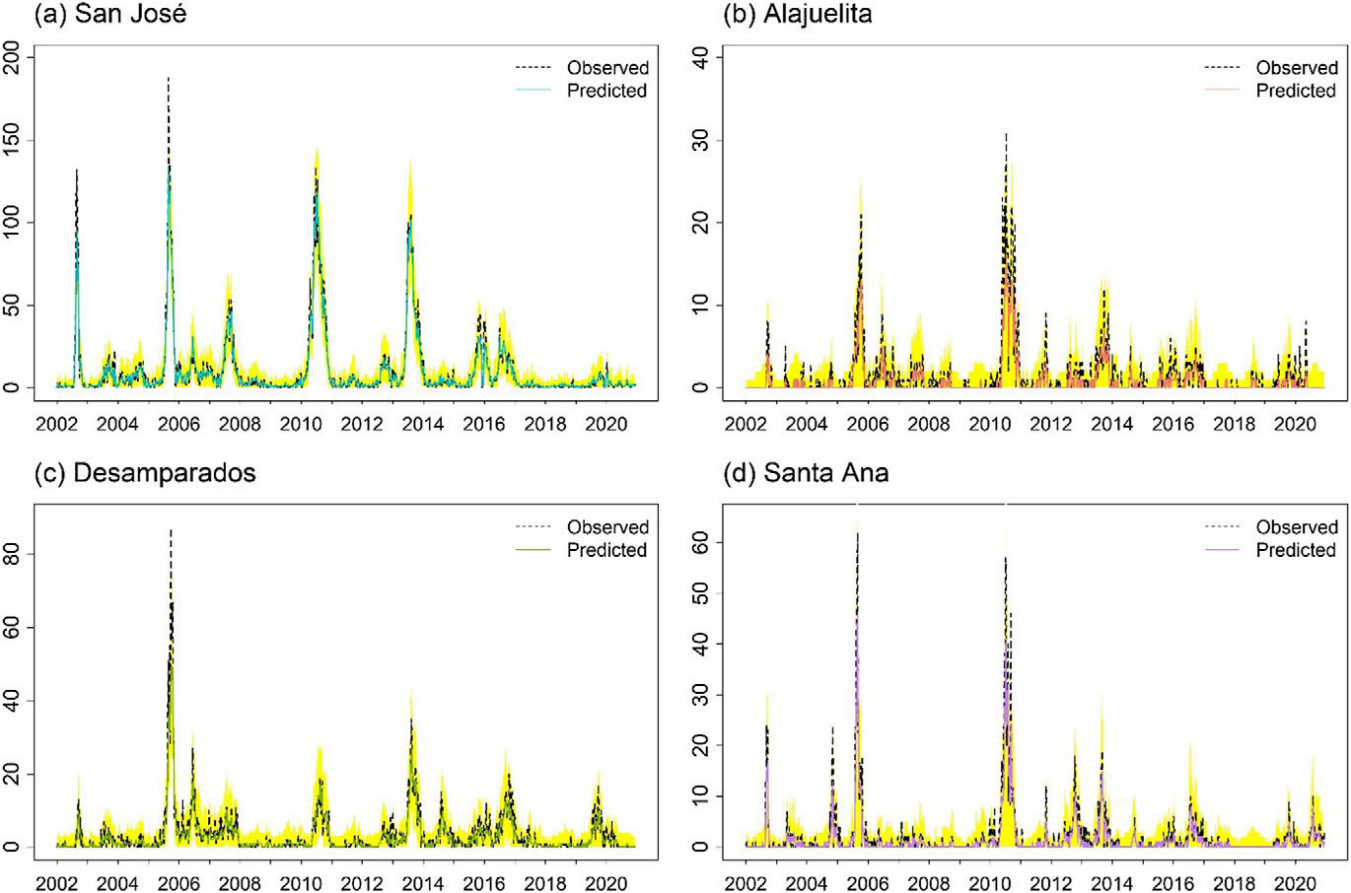
Bayesian predictions and corresponding prediction intervals for weekly dengue case counts in the four municipalities using the spatial ZIGP-INGARCH model with (SIN, COS). Prediction intervals are shown as a shaded yellow band.

For the spatial models, ZIGP with (SIN, COS) yields the lowest pooled RMSE (2.651) and total MSE (28.12). However, differences in both metrics across the top ten models are modest, with most pooled RMSE values ranging from 2.6 to 3.2, corresponding to average weekly errors differing by only about 0.6 dengue cases per municipality. In practice, such models support early warning by flagging weeks in which observed counts exceed high posterior predictive thresholds, such as the 95% prediction interval. The Supplementary Material provides Bayesian estimates for the spatial ZIGP-INGARCH model with (SIN, COS) covariates and corresponding MCMC diagnostic plots, together with parameter estimates for the EE model using the same seasonal specification. Figure 6 presents the weekly observed and predicted dengue case counts for the four municipalities from 2002 to 2020 based on the EE model with (SIN, COS). Figure 7 shows the corresponding prediction plot for the ZIGP-INGARCH model with (SIN, COS), which more effectively accommodates excess zeros and overdispersion while retaining a good fit to outbreak dynamics. The EE model captures the major outbreaks and temporal patterns well, particularly in San José, while the ZIGP-INGARCH model provides improved performance in municipalities with a high proportion of zero weeks, such as Alajuelita and Santa Ana.

Seasonality is captured using sine and cosine terms, which together describe both the magnitude and timing of the annual dengue cycle. For ease of interpretation, these terms can be summarized by an amplitude, reflecting the strength of seasonal variation, and a phase, indicating the week of the year when peak transmission occurs. This representation allows direct comparison of seasonal intensity and timing across municipalities.

Table 4 summarizes the estimated seasonal amplitude and peak timing derived from the EE model, revealing substantial heterogeneity in dengue seasonality across municipalities. Santa Ana exhibits the strongest seasonal variation (amplitude = 0.590), whereas Alajuelita shows a much weaker seasonal signal (0.145). Notably, peak dengue transmission occurs in late September to early October (weeks 39–41) for Desamparados, San Jos’e, and Santa Ana but substantially earlier in late July (week 29) for Alajuelita. These location-specific differences in both the strength and timing of seasonal transmission have direct implications for public health practice. In particular, the earlier peak observed in Alajuelita suggests that vector control and enhanced surveillance efforts may need to be initiated earlier than in other municipalities, while areas with stronger seasonal intensity may require more sustained intervention during peak periods. Together, these findings underscore the importance of tailoring dengue control strategies to local transmission dynamics rather than relying on uniform intervention schedules across regions.

**Table 4.** Seasonal amplitude and peak timing derived from the EE model (period = 52 weeks)

Municipality	Amplitude	Peak week
San José	0.327	39
Alajuelita	0.145	29
Desamparados	0.235	40
Santa Ana	0.590	41

Despite their clear biological relevance, directly including rainfall and temperature as covariates does not always lead to superior short-term predictive performance. Weekly climate measurements are subject to measurement error, spatial aggregation, and heterogeneous lag structures, which can weaken their predictive contribution when incorporated linearly into time series models. Specifically, the four municipalities in this study are geographically proximate and experience similar annual rainfall and temperature cycles, resulting in synchronized seasonal fluctuations in dengue incidence across locations. In this setting, Fourier seasonal factors provide greater explanatory power than the corresponding climatic covariates. In addition, the relationship between climate and dengue incidence is often mediated by unobserved factors such as human behaviour, vector control interventions, and local infrastructure, which are not captured in the available data. Consequently, climate covariates may enhance model interpretability without necessarily yielding substantial gains in forecast accuracy.

In summary, the EE model and the spatial ZIGP-INGARCH model, both incorporating sine and cosine Fourier terms, achieve the best performance across forecast comparison metrics. Although these models differ in how they parameterize seasonality, both consistently identify strong and significant annual cycles in dengue incidence. These results reinforce that Fourier-based representations of annual seasonality effectively capture dengue dynamics in Costa Rica’s Central Valley and highlight the value of incorporating cyclical patterns into spatio-temporal models to support early warning systems and guide municipality-specific vector control. More generally, the appropriate number of seasonal terms depends on regional transmission characteristics and the dominant temporal scales observed in the data [16].

The proposed framework is well-suited for forecasting and early warning applications. Without loss of generality, we outline a natural out-of-sample validation design for the spatial ZIGP-INGARCH model defined in Equations (1)–(2).

To generate a one-step-ahead prediction for location *i* at time 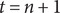 , we first simulate the latent log-intensity 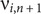 using the dynamic equation given in Equation (2). For each posterior draw of the model parameters 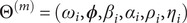 , we compute

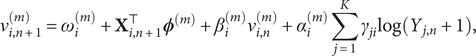

and then map this quantity to the intensity scale via

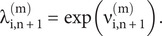

Finally, conditional on 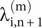 , we draw the one-step-ahead count from the ZIGP model:

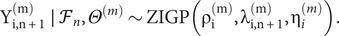



Repeating this procedure for 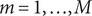 yields a Monte Carlo approximation to the posterior predictive distribution of 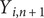 , from which we compute point forecasts and Bayesian prediction intervals. This procedure enables a probabilistic assessment of short-term dengue risk, offering interpretable uncertainty quantification that can inform early warning and situational awareness in dengue surveillance.

## Conclusion

Our analysis reveals clear spatial and temporal heterogeneity in dengue dynamics across municipalities in Costa Rica’s Central Valley. The Poisson-based EE model captures the EE structure and identifies important seasonal patterns, with notable variation in both amplitude and peak timing across municipalities. At the same time, descriptive statistics and prediction results show that overdispersion and excess zeros are substantial features of the data, particularly in Alajuelita and Santa Ana. Addressing these characteristics through the spatial ZIGP-INGARCH and hurdle-INGARCH models enhances predictive performance while complementing the epidemiological interpretation provided by the EE framework.

Importantly, the two model families differ in how forecasts are generated: the EE model relies on conditional means, whereas INGARCH-family models directly produce conditional predictions of case counts. Taken together, these results indicate that no single model is sufficient. The EE model offers valuable insights into the seasonal intensity and timing of dengue transmission, while INGARCH-family models better capture the statistical properties of the data. Integrating insights from both approaches is therefore essential for strengthening early warning systems and guiding locally tailored vector control interventions in Costa Rica.

From a public health perspective, the results highlight the importance of accounting for both spatial interaction and local heterogeneity when modelling dengue transmission. The spatially structured epidemic component reveals how transmission risk propagates across neighbouring locations, while the zero-inflated and overdispersed INGARCH formulations improve the characterization of low-incidence periods and outbreak variability. Together, these features support more reliable short-term risk assessment and enhance the interpretability of early warning signals at the municipal level. In particular, location-specific differences in peak timing suggest that the initiation and timing of surveillance and vector control interventions should be adapted to local transmission patterns rather than implemented uniformly across regions.

From an applied perspective, the proposed framework naturally extends to short-horizon forecasting and early warning systems for dengue surveillance. By allowing model parameters and latent states to update sequentially as new information becomes available, the approach supports timely detection of elevated risk levels and facilitates real-time monitoring at the municipal level. This makes the framework particularly relevant for operational public health decision-making and targeted vector control planning.

## Limitations

This study evaluates model performance using in-sample predictions and does not provide a formal assessment of out-of-sample forecasting accuracy. Consequently, the conclusions primarily emphasize model interpretation and in-sample predictive behaviour rather than real-time predictive performance. In operational dengue surveillance, however, early warning systems rely on short-horizon out-of-sample forecasts that are updated as new weekly data become available. A systematic evaluation of out-of-sample forecasting performance, for example using a rolling-window one-week-ahead prediction scheme over a hold-out period, represents an important direction for future research and would allow the proposed framework to be more directly assessed in an early warning context. Nevertheless, we offer a coherent posterior predictive procedure that provides a probabilistic characterization of short-term dengue risk through one-step-ahead forecasting of dengue counts.

## Supporting information

10.1017/S0950268826101204.sm001 Chen et al. supplementary material Chen et al. supplementary material

## Data Availability

The data have previously been used in the second author’s publications (link) under a different statistical approach. The dataset is openly available in the following GitHub repository: https://github.com/shuwei325/DengueCR_Bayesian_ST_Prediction/blob/main/Data/Casos_Dengue_1993-2021.xlsx.

## Supplementary material

The supplementary material for this article can be found at http://doi.org/10.1017/S0950268826101204.
