# Supplementary material for: Spatio-temporal modelling of dengue counts in the Central Valley of Costa Rica
Source: Epidemiol Infect. 2026 Feb 20;154:e34. doi: 10.1017/S0950268826101204 (PMC13125277; doi:10.1017/S0950268826101204)
Supplement: Chen et al. supplementary material [file S0950268826101204sup001.pdf]

Supplementary materials for “Spatio-temporal modelling of dengue counts in the Central Valley of Costa Rica”

Cathy W.S. Chen[1]*, Shu Wei Chou Chen[2], and Hsiao-Hsuan Liao[1]

[1]Department of Statistics, Feng Chia University, Taichung, Taiwan

[2]School of Statistics & Research Center for Pure and Applied Mathematics, University of Costa Rica, San José, Costa Rica

The supplementary materials support the findings of the main manuscript and include a time plot of weekly dengue case counts for the four municipalities in Costa Rica’s Central Valley, together with the results and interpretation for the two best-performing models.

*Corresponding author: Cathy W.S. Chen, E-mail: chenws@fcu.edu.tw,
https://orcid.org/0000-0001-8727-8168

1 Real Example: Data Description

Figure A1 shows the time series of weekly dengue cases for the four municipalities in Costa Rica's Central Valley. The plots reveal repeated large outbreaks between 2005 and 2014, with dengue activity diminishing substantially after 2018. While the precise timing and magnitude of peaks vary across municipalities, the overall pattern indicates strong temporal clustering of outbreaks followed by a period of reduced transmission.

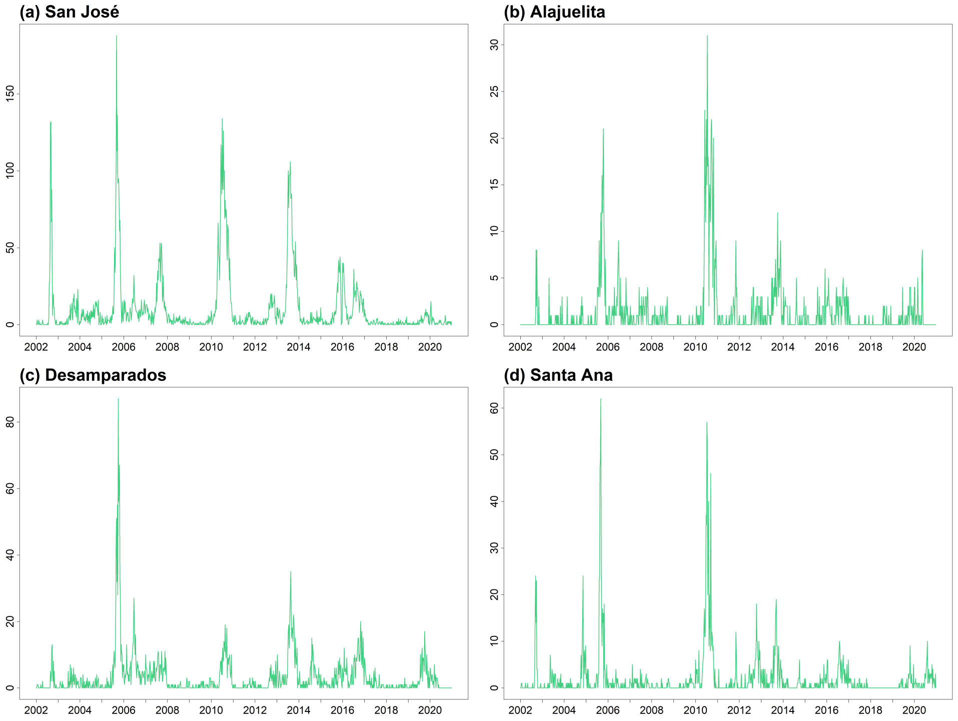

Figure A1: Weekly dengue case counts for the four municipalities in Costa Rica's Central Valley, 2002–2020.

2 Two Best-Performing Models

We discuss the two best-performing models, identified by the smallest pooled root MSE values: the spatial zero-inflated generalized Poisson (ZIGP) integer-valued GARCH (INGARCH) model and the endemic–epidemic (EE) model, both including SIN and COS covariates. When the ZIGP-INGARCH

model is fitted with these covariates:

$$
\begin{aligned}
&Y_{i,t} \mid \mathcal{F}_{t-1} \sim \mathrm{ZIGP}(\rho_i, \lambda_{i,t}, \eta_i), \\
&\nu_{i,t} \equiv \log(\lambda_{i,t}), \\
&\nu_{i,t} = \omega_i + \phi_{i,1}\,\mathrm{SIN}_t + \phi_{i,2}\,\mathrm{COS}_t + \beta_i \nu_{i,t-1} + \alpha_i \sum_{j=1}^{4} \gamma_{ji} \log(Y_{j,t-1} + 1),
\end{aligned}
$$

where $i = 1, \ldots, 4$. Table A1 reports the posterior summaries for the Bayesian estimation of the spatial ZIGP-INGARCH model with SIN and COS seasonal covariates, including the posterior mean, median, standard deviation, and 95% credible intervals for each parameter. Most SIN and COS coefficients have credible intervals that do not include zero, indicating clear seasonal effects. The negative estimates suggest that the seasonal covariates reduce the conditional log-intensity during parts of the annual cycle, while differences across municipalities reflect heterogeneous seasonal patterns.

Figures A2 and A3 present the MCMC diagnostic plots, including the trace plots and autocorrelation functions (ACF). The trace plots fluctuate around stable levels without visible trends, indicating good mixing and convergence of the MCMC chains. The ACF plots decay rapidly toward zero, suggesting low serial dependence and supporting the reliability of the posterior summaries reported in Table A1.

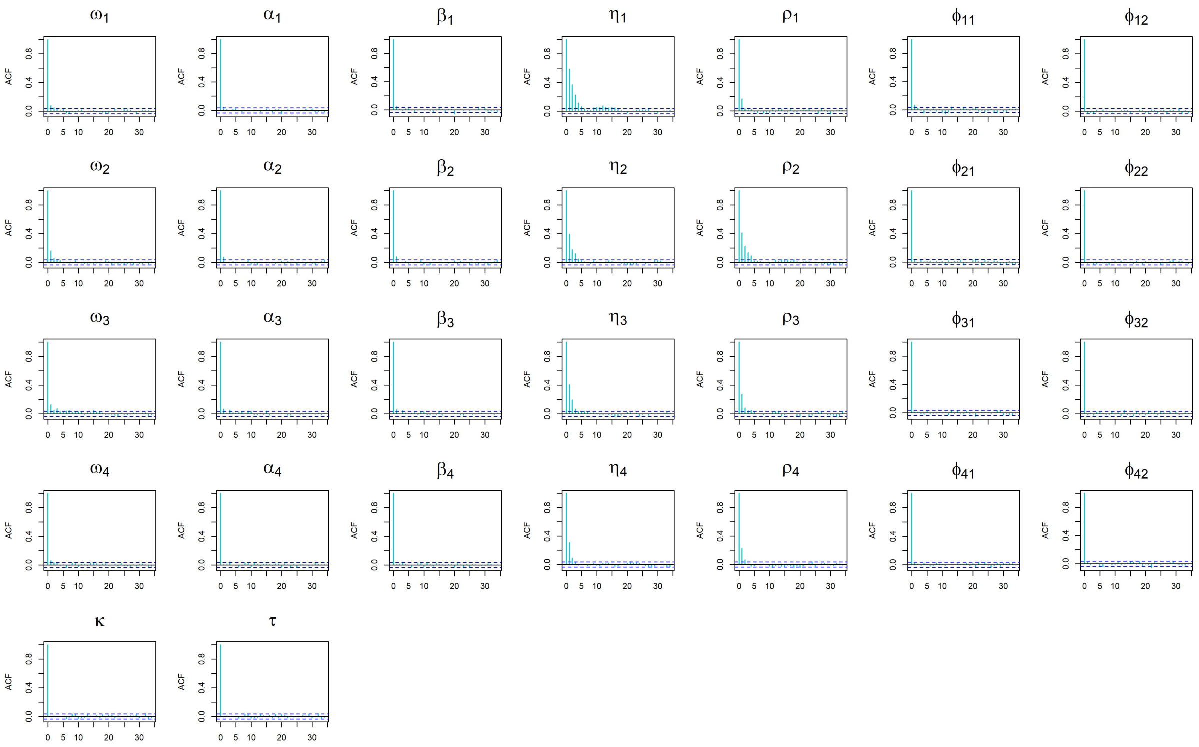

Figure A2: Autocorrelation function (ACF) plots for all parameters in the spatial ZIGP-INGARCH model with (SIN, COS) covariates after the burn-in period.

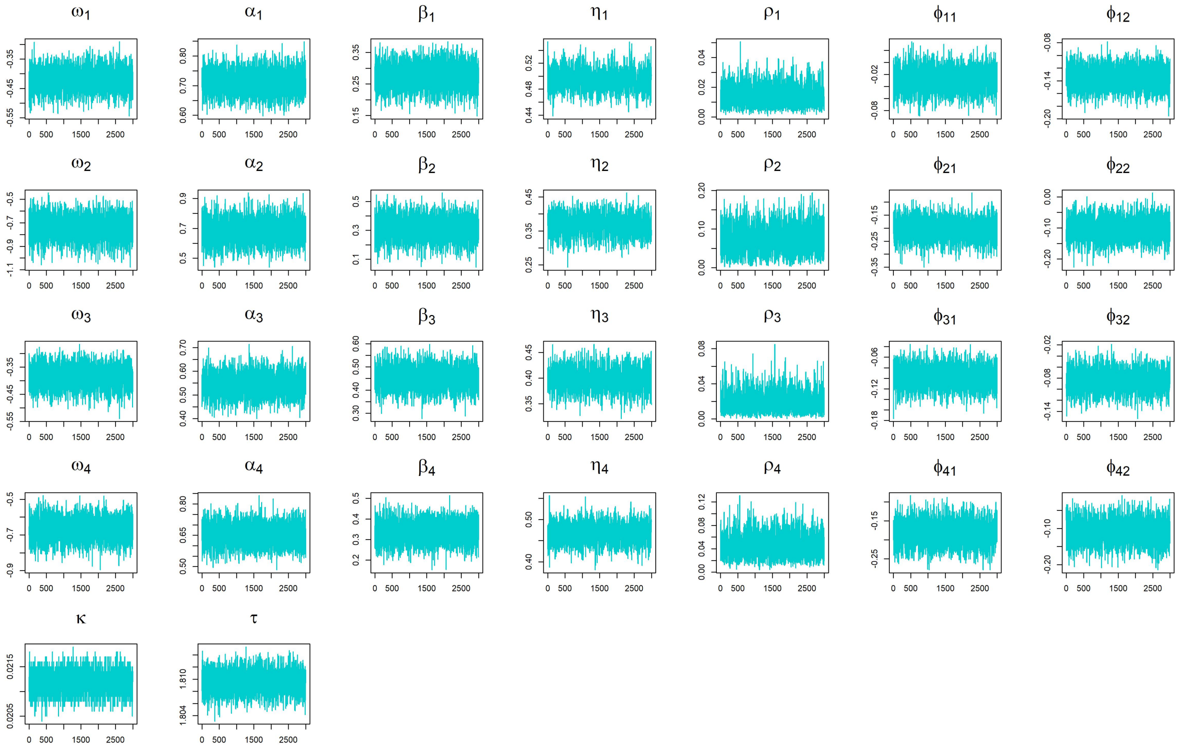

Figure A3: Trace plots for all parameters of the spatial ZIGP-INGARCH model with (SIN, COS) covariates after burn-in.

Table A1: Bayesian estimates for the spatial ZIGP-INGARCH model.

Parameter	Mean	Median	SD	2.5%	97.5%
San José					
ω_1	-0.4204	-0.4209	0.0367	-0.4951	-0.3475
α_1	0.7125	0.7121	0.0380	0.6386	0.7855
β_1	0.2745	0.2748	0.0378	0.1980	0.3489
η_1	0.4954	0.4950	0.0161	0.4648	0.5290
ρ_1	0.0141	0.0131	0.0066	0.0041	0.0290
ϕ_{11} (SIN)	-0.0277	-0.0276	0.0185	-0.0652	0.0077
ϕ_{12} (COS)	-0.1362	-0.1363	0.0168	-0.1685	-0.1037
Desamparados					
ω_2	-0.7382	-0.7352	0.0982	-0.9337	-0.5530
α_2	0.6787	0.6777	0.0766	0.5305	0.8301
β_2	0.3155	0.3161	0.0772	0.1633	0.4650
η_2	0.3673	0.3677	0.0296	0.3062	0.4242
ρ_2	0.0747	0.0712	0.0351	0.0151	0.1496
ϕ_{21} (SIN)	-0.2044	-0.2035	0.0384	-0.2805	-0.1304
ϕ_{22} (COS)	-0.1028	-0.1026	0.0330	-0.1680	-0.0392
San José					
ω_3	-0.3860	-0.3843	0.0400	-0.4677	-0.3074
α_3	0.5427	0.5426	0.0457	0.4544	0.6328
β_3	0.4533	0.4539	0.0458	0.3638	0.5420
η_3	0.3979	0.3978	0.0210	0.3553	0.4395
ρ_3	0.0189	0.0171	0.0111	0.0036	0.0446
ϕ_{31} (SIN)	-0.0974	-0.0972	0.0197	-0.1376	-0.0594
ϕ_{32} (COS)	-0.0838	-0.0834	0.0185	-0.1210	-0.0482
Santa Ana					
ω_4	-0.6700	-0.6684	0.0617	-0.7942	-0.5528
α_4	0.6536	0.6526	0.0508	0.5551	0.7537
β_4	0.3360	0.3374	0.0509	0.2365	0.4327
η_4	0.4671	0.4677	0.0230	0.4224	0.5106
ρ_4	0.0447	0.0422	0.0185	0.0146	0.0879
ϕ_{41} (SIN)	-0.1818	-0.1817	0.0306	-0.2417	-0.1208
ϕ_{42} (COS)	-0.1086	-0.1081	0.0310	-0.1702	-0.0477
κ	0.0212	0.0212	0.0002	0.0208	0.0216
τ	1.8095	1.8095	0.0017	1.8061	1.8129

Notes: SD = posterior standard deviation. SIN and COS are seasonal covariates with period 52 weeks.

The endemic–epidemic (EE) model

We briefly recall the EE count time-series model used in the main text. Let $Y_{i,t}$ denote the reported cases in municipality i at week t and $\mathcal{F}_{t-1}$ the information set up to week $t-1$. The conditional mean $\mu_{i,t}$ is decomposed into an endemic component $\nu_{i,t}$, an autoregressive component $\psi_i Y_{i,t-1}$ capturing within-municipality persistence, and a spatio-temporal epidemic component $\phi \sum_{j \neq i} w_{ij} Y_{j,t-1}$ representing transmission from neighboring municipalities. Seasonality in $\nu_{i,t}$ is modeled using Fourier terms SIN_t and COS_t.

$$\begin{aligned}
&Y_{i,t} \mid \mathcal{F}_{t-1} \sim \mathrm{Poisson}\big(\mu_{i,t}\big), \\
&\mu_{i,t} \;=\; \nu_{i,t} \;+\; \psi_i \, Y_{i,t-1} \;+\; \phi \sum_{j \neq i} w_{ij} \, Y_{j,t-1}, \\
&\nu_{i,t} \;=\; \exp\Big(\alpha_i^{(\nu)} \;+\; \beta_{\mathrm{SIN},i}^{(\nu)} \, \mathrm{SIN}_t \;+\; \beta_{\mathrm{COS},i}^{(\nu)} \, \mathrm{COS}_t\Big), \\
&\psi_i \;=\; \exp\big(\alpha_i^{(\psi)}\big), \qquad \phi \;=\; \exp\big(\alpha^{(\phi)}\big), \quad i = 1, \cdots, 4.
\end{aligned} \tag{1}$$

The EE model can be specified with either a Poisson or a negative binomial distribution. In our analysis, the negative binomial specification produced a higher MSE than the Poisson; therefore, we report only the Poisson-based EE model in Eq. (1).

Table A2 reports the parameter estimates for the EE model with seasonal covariates $(\mathrm{SIN}, \mathrm{COS})$. The epidemic autoregression parameters ψ_i measure the contribution of cases from the same municipality in the previous week, with larger values indicating stronger temporal persistence. San José shows the strongest persistence ($\psi_{\mathrm{SJ}} = 0.882$), whereas Alajuelita has the weakest ($\psi_{\mathrm{A}} = 0.412$). The neighbourhood effect parameter ϕ captures cross-municipality transmission; its small estimate ($\phi = 0.049$) indicates relatively weak spatial spillover. The endemic baseline terms $\exp\{\alpha_i^{(\nu)}\}$ represent the background component of $\nu_{i,t}$ when seasonal covariates are zero. San José has the highest baseline (0.997), followed by Desamparados (0.505), while Alajuelita has the lowest (0.133).

Table A2: Parameter estimates for the EE model with (SIN, COS) covariates.

Municipality	ψ_i	SE	$\exp\{\alpha_i^{(\nu)}\}$	SE	$\exp\{\beta_{SIN,i}^{(\nu)}\}$	SE	$\exp\{\beta_{COS,i}^{(\nu)}\}$	SE
San José	0.882	0.010	0.997	0.054	0.721	0.052	1.002	0.073
Alajuelita	0.412	0.028	0.133	0.020	0.946	0.193	0.875	0.168
Desamparados	0.662	0.019	0.505	0.038	0.791	0.079	1.021	0.097
Santa Ana	0.744	0.021	0.488	0.028	0.562	0.044	1.136	0.086
ϕ (Neighbourhood effect): 0.049 (SE = 0.003)								

Seasonal amplitude and peak timing. Seasonality is modelled through the sine and cosine covariates, $\beta_{\text{SIN},i}^{(\nu)}$ and $\beta_{\text{COS},i}^{(\nu)}$. For interpretability, these coefficients are reparameterised into amplitude–phase form. The seasonal amplitude and phase for municipality i are computed as

$$A_i = \sqrt{\beta_{\text{SIN},i}^2 + \beta_{\text{COS},i}^2}, \qquad \varphi_i = \operatorname{atan2}\left(-\beta_{\text{COS},i}, \beta_{\text{SIN},i}\right).$$

Here, A_i measures the magnitude of seasonal variation, while φ_i determines the timing of the seasonal peak. The corresponding peak week (with period $S = 52$ weeks) is given by

$$\text{week}_i = \{\varphi_i \bmod 2\pi\}\frac{S}{2\pi}.$$

The resulting amplitude and peak timing estimates are reported in Table 4 in the main text.
